# Comparing the Metabolic Characteristics of Hyacinth Bean (*Lablab purpureus* L.) Seeds from Five Local Varieties by UHPLC-QE HF HRMS

**DOI:** 10.3390/foods14111939

**Published:** 2025-05-29

**Authors:** Li Yu, Zhiwu Huang, Luzhao Pan, Hengyu Meng, Weimin Zhu, Jun Yan

**Affiliations:** Key Laboratory of Protected Horticulture Technology, Shanghai Academy of Agricultural Sciences, Shanghai 201403, China; yuhe_5@126.com (L.Y.);

**Keywords:** hyacinth bean seeds, metabolic characterization, UHPLC-QE HF HRMS, antioxidant activity

## Abstract

Hyacinth bean seeds are a good source of vegetable protein and have great potential for medicinal development. However, their metabolic characteristics are unclear. Therefore, in this study, we conducted non-targeted metabolomics research on hyacinth bean seeds from local varieties using ultra-high-performance liquid chromatography combined with high-field quadrupole orbital trap high-resolution mass spectrometry (UHPLC-QE HF HRMS) and evaluated their antioxidant properties. A total of 745 metabolites were identified, including many bioactive medicinal compounds such as chikusetsusaponin IVa, pipecolic acid, and genistin. The seed coat color and origin of hyacinth bean seeds have significant impacts on their metabolic characteristics. Compared with the other four hyacinth beans, the Chongming white hyacinth bean (SCLW) has a higher medicinal value, with glycitin, finsenoside Ro, diferuloyl glycerol, isopongflavone, procyanidin B2, and pratensein speculated to be its characteristic metabolites. DPPH and FRAP assays showed that the antioxidant activity of SCLW was significantly higher than that of the other four hyacinth bean seeds, and 11 metabolites related to antioxidant activity were identified. These findings enrich our knowledge of the metabolites in hyacinth bean seeds, which is of great significance for hyacinth bean cultivation according to local conditions and for the improvement of variety quality.

## 1. Introduction

In the face of an increasingly severe global food crisis, exploring and utilizing potential crop resources is of crucial importance for ensuring food security and promoting sustainable agricultural development [1]. Hyacinth bean (*Lablab purpureus* L.) is an important crop and a common vegetable variety. Its origin can be traced back to 1500 BC, and it is now widely cultivated in tropical, subtropical, and temperate regions [2]. In China, the hyacinth bean has a long history of cultivation and is distributed over a vast area ranging from the south to the north. Hyacinth bean is a multi-functional leguminous plant. It contains a variety of amino acids essential for the human body and is abundant in vitamins, minerals, and dietary fiber. The seeds and pods are rich in protein, with a content of up to 20–28%, making it an excellent source of plant-based protein [3]. In the theory of traditional Chinese medicine, hyacinth bean is regarded as an ingredient with medicinal value. It has the effects of invigorating the spleen and removing dampness, harmonizing the middle-jiao and relieving summer heat, replenishing qi, etc. It can be used to treat symptoms such as weakness of the spleen and stomach, poor appetite, and vomiting and diarrhea due to summer dampness [4]. Modern pharmacological studies have shown that the hyacinth bean is rich in various phytochemicals, such as aloinoside, chrysophanol, rheinphenol, etc.; it also contains components such as antibiotics, alkaloids, and flavonoids [5,6]. The plant can be successfully used to treat diarrhea and reduce blood sugar, as well as for its anti-cancer properties [5,7].

With the improvement in people’s health awareness and the increasing concern for food safety, the demand for nutritious, eco-friendly, and healthy foods is constantly on the rise. As a nutritious ingredient with medicinal value, hyacinth bean meets the expectations that the modern consumer has of healthy foods, which grants it broad market prospects. However, current research on hyacinth bean mainly focuses on its medicinal mechanisms [8,9,10] and processing and utilization [11,12,13]. Studies on hyacinth bean resources in the Indian region have shown that variety and geographical location have a significant impact on the plant’s nutrient content [14,15]. China has a long history of cultivating the hyacinth bean, and there are many excellent local varieties, such as the white hyacinth bean in Chongming District, Shanghai, the red hyacinth bean in Pudong District, Shanghai, the white hyacinth bean in Yunnan Province, and the white hyacinth bean in Sichuan Province. Understanding the quality characteristics of hyacinth beans from different regions is helpful for the development of hyacinth bean sources, as well as for providing a theoretical basis for variety improvement. To date, no studies have comprehensively analyzed the metabolites in hyacinth beans.

Comprehensive and accurate analytical techniques are crucial for elucidating the quality characteristics of hyacinth beans. Non-targeted metabolomics is a comprehensive analytical method that aims to detect and identify as many metabolites as possible in a given sample. Next-generation metabolomics features higher resolution, stronger mass stability, and precision. The standards for metabolite identification are aligned with those of international journals (MSI), enabling the acquisition of more complete information from samples. Meanwhile, level 1 identification achieves a high identification accuracy rate (approximately 98%). The metabolomics approach has not yet been applied to the analysis of the nutritional components of hyacinth beans. Therefore, in this study, UHPLC-QE HF HRMS technology was utilized to analyze the metabolic characteristics of five local varieties of hyacinth bean. Differential metabolites were analyzed based on orthogonal partial least squares discriminant analysis (OPLS-DA) and analysis of variance (ANOVA). The antioxidant activities of the different hyacinth bean varieties were studied, and the metabolites related to these antioxidant activities were identified.

## 2. Materials and Methods

### 2.1. Plant Material

**Figure 1:**
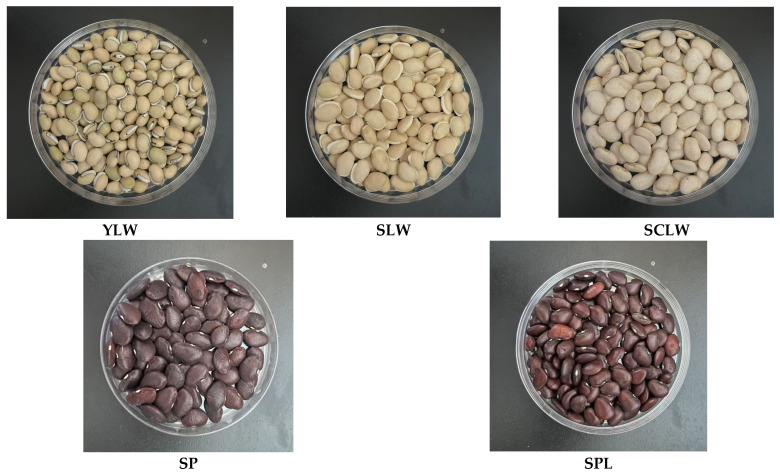
Five local hyacinth bean varieties in China.

In this study, the following Chinese varieties of hyacinth bean were used: white hyacinth bean (chongming white hyacinth bean); two red hyacinth bean varieties (Jiading Zhuxuebian and Pudong red hyacinth bean) from Shanghai; white hyacinth bean from Chongxiong, Yunnan Province; and white hyacinth bean from Chengdu, Sichuan Province (Table S1, Figure 1). These hyacinth beans were collected from local planting areas using the five-point sampling method, with a total of 500 g sampled for each variety. Before the analysis, the hyacinth bean samples were freeze-dried and ground into powder. The powder was stored at −80 °C until the metabolomic analysis.

### 2.2. UHPLC-QE HF HRMS Analysis

Hyacinth bean powder (50 mg ± 1 mg) was mixed with 1000 μL of extraction solution (MeOH:ACN:H_2_O, 2:2:1 (*v*/*v*)) containing isotope-labeled internal standards. The mixed solution was vortexed for 30 s. Then, the mixed samples were homogenized (35 Hz, 4 min) and sonicated for 5 min in a 4 °C water bath; this step was repeated three times. The samples were incubated for 1 h at −40 °C to precipitate the proteins. Then, the samples were centrifuged at 12,000 rpm for 15 min at 4 °C. The supernatants were filtered with a 0.22 μm hydrophilic PTFE syringe filter (SCAA-114, ANPEL, Shanghai, China), and the filtrate was maintained at −20 °C until testing. Three replicates were performed on each hyacinth bean sample. The quality control (QC) sample was prepared by mixing an equal aliquot of the supernatant of the samples.

Liquid chromatography–tandem mass spectrometry (LC-MS/MS) analysis was carried out using an ultra-high-performance liquid chromatography system (Thermo Fisher Scientific Inc., Vanquish, MA, USA) combined with a Phenomenex Kinetex C18 chromatographic column (2.1 mm × 100 mm; particle size 2.6 μm) and an Orbitrap Exploris 120 mass spectrometer (Orbitrap mass spectrometer, Thermo Fisher Scientific Inc.). Mobile phase A was an aqueous solution containing 0.01% acetic acid; mobile phase B was a mixed solution of isopropanol and acetonitrile (volume ratio 1:1). Column temperature was set at 25 °C, the temperature of the autosampler was 4 °C, and the injection volume was 2 μL. The Orbitrap Exploris 120 mass spectrometer collected primary and secondary mass spectrometry data; control software (Xcalibur, version: 4.4, Thermo) was also used. The detailed parameters are as follows: sheath gas flow rate: 50 Arb; Aux gas flow rate: 15 Arb; capillary temperature: 320 °C; full ms resolution: 60,000; MS/MS resolution: 15,000; collision energy: SNCE 20/30/40; spray voltage: 3.8 kV (positive) or −3.4 kV (negative). Isotope internal standards were used for the quality control of data collection stability.

### 2.3. Identification and Quantification of Non-Volatile Metabolites

After the raw data were converted into the mzXML format using ProteoWizard software (3.0.9134), a jointly developed R package was used for metabolite identification. The databases used were BiotreeDB (V3.0, standard substance library) and BT-Plant (V1.1, plant-specific library). The identification results were annotated with the identification levels of the metabolites by referring to the Metabolomics Standards Initiative (MSI). The annotations are as follows: Level 1: The metabolite in the sample matches the MS^1^, MS^2^, and RT of the standard substance. Level 2: The metabolite in the sample matches both the MS^1^ and MS^2^ in the public database. Level 3: The metabolite in the sample matches the MS^1^, MS^2^, and predicted RT in the theoretical database. Level 4: Unknown compound.

### 2.4. Analysis of Antioxidant Capacity

Sample extraction: Each hyacinth bean sample was grounded with liquid nitrogen. Then, 0.5 g of the powder was added to a shaking incubator containing 5 mL of 80% ethanol for 6.5 h at room temperature. After centrifugation at 10,000 rpm for 10 min, the supernatants were collected. Powdered samples (0.5 g) were extracted in 4.5 mL of 80% ethanol for 6 h at room temperature in a shaking incubator, followed by centrifugation at 10,000 rpm for 10 min. The supernatants were carefully collected and used for DPPH and FRAP assays.

DPPH assay: DPPH scavenging activity was evaluated as previously described [16]. Sample extract (1.0 mL), DPPH solution (2 mL 2 × 10^−4^ mol/L), and absolute ethanol (4.0 mL) were mixed and incubated in the dark at room temperature for 30 min. Then, absorbance was determined at 515 nm wavelength using a spectrophotometer. The scavenging rate was calculated according to the following formula: Scavenging rate = [1 − (A1 − A2)/A0] × 100%. A0 is the absorbance when absolute ethanol is used to replace the sample; A1 is the absorbance when the sample and DPPH· are added; and A2 is the absorbance when absolute ethanol is used to replace DPPH.

FRAP assay: A ferric ion-reducing antioxidant power (FRAP) assay was performed according to the method reported by Pulido et al. (2000) [17] with some modifications. Sample extract (30 μL) was added to the FRAP reaction solution and diluted to 10 mL. After incubation for 30 min at 37 °C, absorbance was measured at 593 nm wavelength using a microplate reader. A standard curve was drawn using a standard solution (20 µmol/mL FeSO_4_). The FRAP reaction solution was made by mixing 0.3 μmol/mL acetate buffer (pH 3.6), 0.02 mol/L TPTZ solution (prepared with a 40 mmol/L HCl solution), and 0.02 mol/L FeCl_3_ 6H_2_O at a ratio of 9:1:1. The antioxidant capacity of the sample was calculated by the Fe^2+^ concentration according to the standard curve.

### 2.5. Statistical Analysis

Principal component analysis (PCA) and orthogonal partial least squares discriminant analysis (OPLS-DA) were carried out using SIMCA software 14. The differences in metabolite contents were assessed via one-way analysis of variance (ANOVA) with Duncan’s multiple range test using SPSS 25.0 software.

## 3. Results and Discussion

### 3.1. Metabolic Properties of Hyacinth Beans Seed

**Figure 2:**
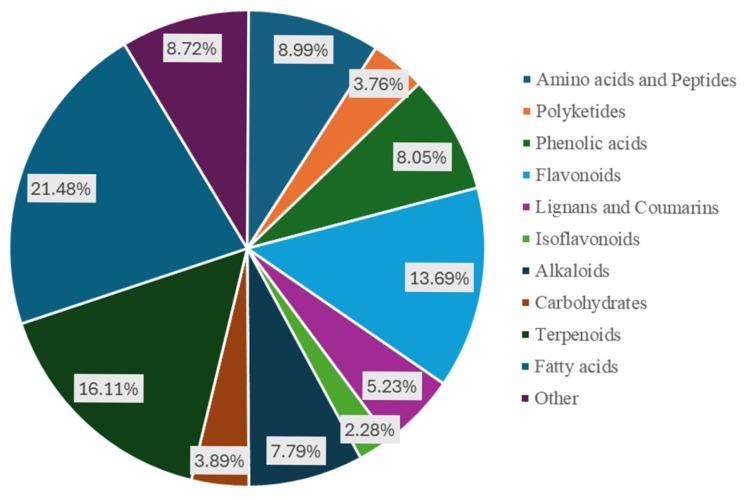
Classification of the 745 identified metabolites.

We used a non-targeted metabolomics analysis method based on UHPLC-QE HF HRMS to conduct metabolomics analysis on hyacinth bean seeds from five local varieties (designated as YLW, SLW, SCLW, SLP, and SP). The identification results were annotated according to the Metabolomics Standards Initiative (MSI) [18] to determine the level of the metabolites identified. A total of 33,802 metabolites were detected in the five hyacinth bean varieties (Table S2); of these, 423 metabolites were identified as level 1, and 322 metabolites were identified as level 2. The 745 metabolites identified were classified into 11 categories, including phenolic acids (8.05%, 34 at level 1), flavonoids (13.69%, 56 at level 1), fatty acids (21.48%, 117 at level 1), amino acids and their derivatives (8.99%, 28 at level 1), alkaloids (7.79%, 25 at level 1), carbohydrates (3.89%, 27 at level 1), lignans and coumarins (5.23%, 19 at level 1), terpenes (16.11%, 25 at level 1), polyketides (3.76%, 19 at level 1), and others (8.72%, 26 at level 1) (Figure 2). Therefore, flavonoids, terpenes, and fatty acids are the main metabolites in hyacinth bean seeds.

In this study, a large number of secondary metabolites were detected in the hyacinth bean, some of which have been proven to have medicinal functions. For example, the polyketide substance emodin has a broad-spectrum anti-cancer effect [19], and genistin, which is a major isoflavone in legumes, has antibacterial and anti-cancer effects [20]. Pipecolic acid and polysaccharide have been proven to have antidiarrheal effects [21]. According to previous reports, a total of 27 triterpenoid saponins have been isolated in hyacinth beans. In this study, 76 triterpenoid saponins were detected (26 at level 1), among which chikusetsusaponin IVa is a representative saponin with anti-inflammatory [22], antioxidant [23], and antidiabetic properties [24]. Yue et al. (2021) [25] isolated seven flavonoids from hyacinth beans, namely kaempferol-3-O-sophoroside, quercetin 3-glucoside, kaempferol-3-O-robinobioside, Kaempferol-3-O-rutinoside, isorhamnetin 3-O-neohesperidoside, kaempferol-3-O-galactoside, and quercetin 3-rutin. Among them, kaempferol-3-O-sophoroside, kaempferol 3-O-rutinoside, isorhamnetin 3-O-neohesperidoside, and kaempferol 3-O-galactoside—flavonoids with antioxidant and hypoglycemic effects—were also detected in this study [26,27,28]. Hyacinth beans were previously reported to contain 0.62% oil, mainly including palmitic acid, linoleic acid, stearic acid, etc. [29]. The fatty acids detected in this study include various unsaturated fatty acids, such as linolenic acid and arachidonic acid, which are beneficial for reducing cholesterol and preventing cardiovascular and cerebrovascular diseases [30].

**Figure 3:**
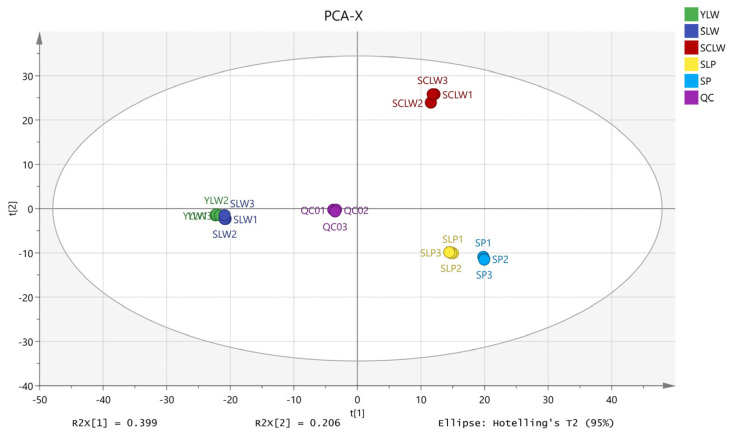
PCA score plots of five varieties of hyacinth bean seeds.

Principal component analysis (PCA) is helpful for understanding the overall metabolic differences between different groups and the degree of variation between samples within the same group [31]. Here, the R2X (cum) of the PCA model was 0.920 and the Q2 (cum) was 0.864 (Figure 3), indicating that the model performed well. The five hyacinth bean varieties have independent regions on the PCA score plot, indicating that there are differences in their metabolite profiles. As shown in Figure 2, the five hyacinth bean varieties are distributed in three regions. Among them, SCLW is located on the bottom right, SLP and SP are located on the top right, and the white hyacinth beans from Yunnan (YLW) and Sichuan (SLW) are located on the left. These results show that the metabolite profiles vary not only according to the different seed coat colors but also depending on the geographical origin. Studies on sesame [32], rice [33], and quinoa [34] have all reported similar conclusions, indicating that both genetic factors and environmental differences can influence the metabolic profiles of plants. In this study, SCLW, YLW, and SLW are white hyacinth beans. SCLW comes from Chongming District, Shanghai, which has a significantly lower altitude than Sichuan and Yunnan. The climate of the three regions is also different. The impact of the environment on plant metabolites is a complex process, and more experiments are required to elucidate the degree of influence that different environmental factors may exert.

### 3.2. Identification of Differential Metabolites Among Different Hyacinth Bean Seeds

**Figure 4:**
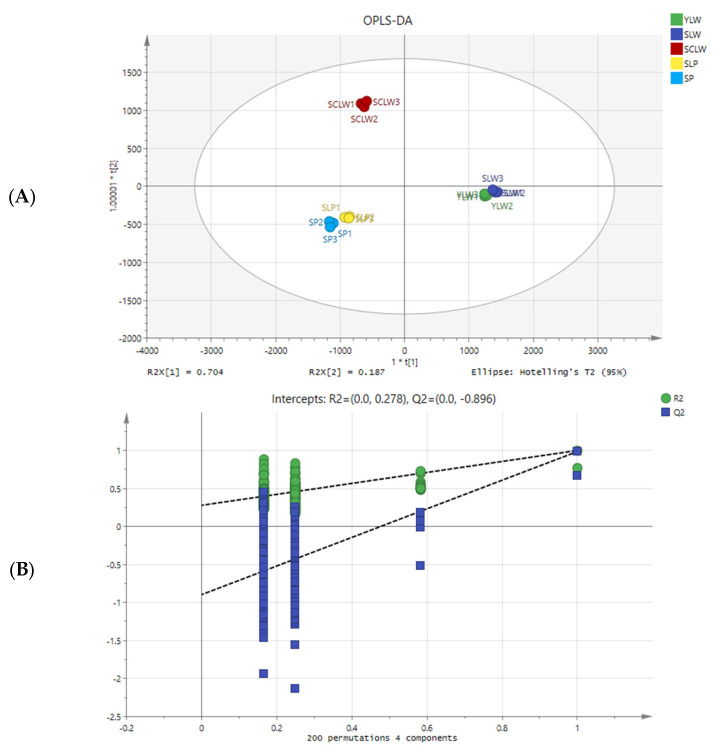
OPLS-DA score plots (**A**) and the two hundred permutation tests (**B**) of five hyacinth bean varieties seeds.

In this study, OPLS-DA and ANOVA were used to identify the differences in metabolites between hyacinth bean varieties. In the OPLS-DA score plot, the distribution of the five hyacinth bean varieties was similar to PCR analysis, with R2 x cum, R2 y cum, and Q2 cum values of 0.987, 0.996, and 0.987, respectively (Figure 4A). Then, 200 permutation tests were conducted to determine whether the supervised OPLS-DA model was overfitting. The original points of R2 and Q2 on the right are higher than the values of R2 and Q2 arranged on the left, indicating that the OPLS-DA model is reliable (Figure 4B) [35]. *p*-value < 0.05 and a variable importance projection (VIP) ≥ 1 were used as the criteria to screen for significantly different metabolites in pairwise comparisons. There were 175 significantly different metabolites (110 of them upregulated) in YLW vs. SLW; 380 (165 upregulated) in YLW vs. SCLW; 352 (172 upregulated) in YLW vs. SLP; 395 (168 upregulated) in YLW vs. SP; 389 (141 upregulated) in SLW vs. SCLW; 376 (163 upregulated) in SLW vs. SLP; 396 (153 upregulated) in SLW vs. SP; 269 (179 upregulated) in SCLW vs. SLP; 266 (148 upregulated) in SCLW vs. SP; and 94 (24 upregulated) in SLP vs. SP (Table 1).

As shown in Table 1, the differential metabolites in the hyacinth bean variety seeds are mainly secondary metabolites. The classification of differential metabolites shows that white hyacinth beans have higher levels of phenolic acids, coumarins, and terpenes, while red hyacinth beans have higher levels of flavonoids. According to previous reports, higher levels of flavonoids were also detected in black sesame and black rice [32,33]. The classification of significantly different metabolites between Chongming white hyacinth beans and white hyacinth beans from Sichuan and Yunnan shows that the former variety contains higher levels of amino acids, fatty acids, and flavonoids. Chongming white hyacinth bean is not only rich in nutritional components, but it also contains high levels of medicinal ingredients. The classification of significantly different metabolites between YLW and SLW shows that YLW contains higher levels of flavonoids and terpenes, while that between SLP and SP shows that SP has a higher content of amino acids, phenolic acids, flavonoids, terpenes, and alkaloids. Shanghai, Sichuan, and Yunnan are located in different geographical regions, which translates into disparities in climate and soil conditions. Verma and Shukla (2015) [36] pointed out that environmental factors such as temperature, light, and water are crucial determinants of biosynthesis and flux in plant secondary metabolites.

### 3.3. Characteristic Metabolites in Chongming White Hyacinth Bean(SCLW) Seeds

**Figure 5:**
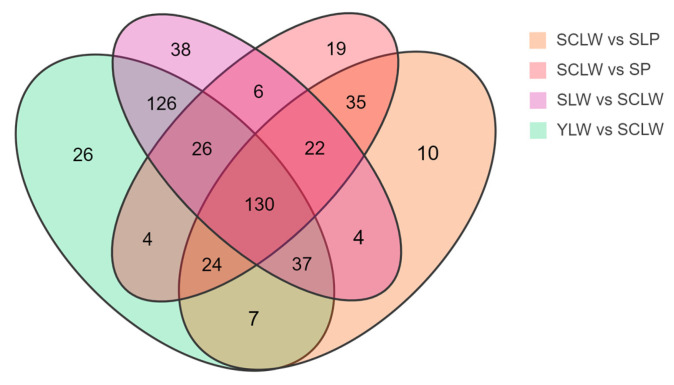
Venn diagram showing the numbers of different metabolites among five varieties of hyacinth bean seeds.

In the PCA and OPLS-DA score plots, SCLW is distributed in independent regions and is significantly distinguished from the other four varieties. We constructed a Venn diagram of the differential metabolites in SCLW vs. SLP, SCLW vs. SP, SCLW vs. YLW, and SCLW vs. SLW (Figure 5). There are 130 significantly overlapping different metabolites in these four pairwise comparisons, among which the number of upregulated metabolites in SCLW is about twice that of downregulated metabolites. In the comparisons of SCLW vs. SLP and SCLW vs. SP, there were 50 and 62 metabolites, respectively, with fold changes greater than 10. These metabolites were mainly flavonoids, phenolic acids, lignans, coumarins, and terpenes, among which there were 36 overlapping metabolites (14 of which were upregulated) (Table S3). The downregulated metabolites were mainly flavonoids related to anthocyanin metabolism. Ginsenoside Ro, glycitin, and ghikusetsaponin IVa, which are common and have been identified as biologically active metabolites in legumes [4], were significantly upregulated in SCLW. In the comparisons of SCLW vs. YLW and SCLW vs. SLW, there were 101 and 107 metabolites, respectively, with fold changes greater than 10. These metabolites were mainly flavonoids, phenolic acids, alkaloids, and terpenes, and 73 (47 upregulated) were overlapping (Table S4). The upregulated substances were mainly flavonoids, and others were bioactive metabolites commonly identified in leguminous plants, such as glycitin, soyasaponin Ba, and ginsenoside Ro [4]. These results show that Chongming white hyacinth bean seeds have a higher medicinal value than the seeds of other local hyacinth bean varieties.

Six metabolites with fold changes greater than 10 overlapped in the four comparisons; these were glycitin (up), ginsenoside Ro (up), diferuloyl glycerol (down), isopongflavone (down), procyanidin B2 (down), and pratensein (down). Procyanidin B2 and pratensein are involved in anthocyanin metabolism. Glycitin is a type of soy isoflavone that has anti-osteoporosis and heart-protective effects [37], while ginsenoside Ro is a triterpene saponin with anti-inflammatory and anti-diabetic effects [38,39]. These metabolites are speculated to be characteristic metabolites of Chongming white hyacinth bean seeds. However, the sample size used in this study was relatively small. Thus, more samples need to be analyzed in the future to confirm this hypothesis.

### 3.4. Variation in Antioxidant Activities of Different Hyacinth Bean Seeds

**Figure 6:**
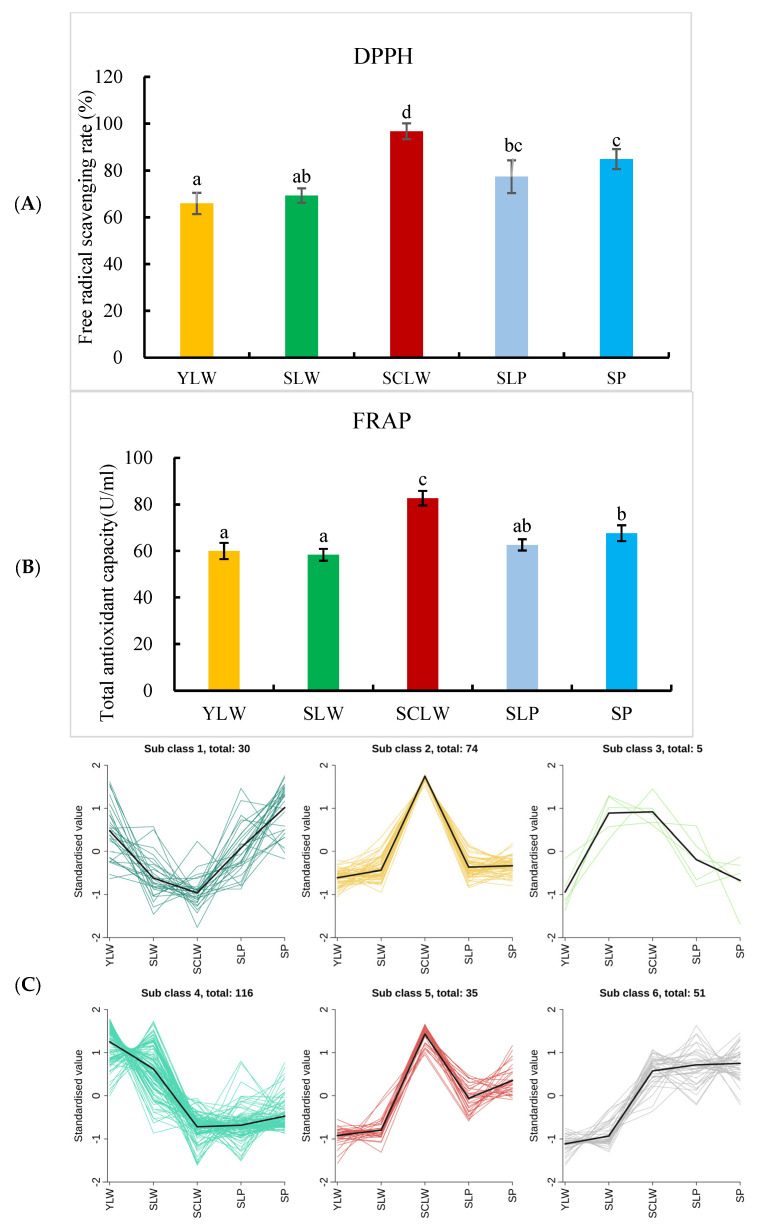
The K-mean clustering analysis of different metabolites and antioxidant activities of the different hyacinth bean varieties seeds. (**A**) DPPH free radical scavenging rate of the five hyacinth bean seeds (%); (**B**) FRAP, ferric reducing antioxidant potential (U/mL); (**C**) K-mean clustering analysis of different metabolites in YLW, SLW, SCLW, SPL, and SP. Note: Lowercase letters indicate significant differences, *p* < 0.05.

To reveal the potential correlation between the metabolites in hyacinth bean seeds and their antioxidant activities, we evaluated the antioxidant activities of the five local hyacinth bean varieties using the DPPH and FRAP methods (Figure 6A,B). Among them, SCLW had the highest antioxidant activity, while SP had significantly higher antioxidant activity than YLW and SLW. K-means clustering analysis of the relative content of differential metabolites yielded six groups (Figure 6C). Among them, groups 2 and 5 showed similar trends in terms of the change in antioxidant activity in the five hyacinth bean varieties. Group 2 had 74 metabolites (Table S5), including 5 phenolic acids, 18 flavonoids, 11 terpenes, 4 alkaloids, 2 coumarins, and 1 isoflavone. Group 5 had 35 metabolites, including 1 phenolic acid, 4 flavonoids, 1 isoflavone, 2 terpenes, and 1 alkaloid (Table S5). Sinapic acid, cynarine, camelliain B, grosvenorine, kaempferol 7-O-β-D-glucopyranoside, ternatumosis II [40,41], verminoside [42], methylophiopogonanone A [43], dihydroferulic acid [44], keracyanin [45], and aloenin A [46] have proven free radical-scavenging and antioxidant effects. The relative concentration of these 11 metabolites in SCLW seeds was significantly higher than that in the other four hyacinth beans. It is speculated that these 11 metabolites are key antioxidants in hyacinth bean seeds. In this study, a change was found in the antioxidant activity of the five hyacinth bean varieties analyzed. In order to study the antioxidant activity of hyacinth beans in more depth, in subsequent research, we will use methods that involve biologically relevant free radicals, such as the ORAC (Oxygen Radical Absorbance Capacity) method, to evaluate the antioxidant activity of the seeds and seed extracts of more hyacinth bean varieties. Thus, we aim to provide a theoretical basis for the full and effective utilization of hyacinth beans.

## 4. Conclusions

This study analyzed the metabolic profile of hyacinth bean seeds using the UHPLC-QE HF HRMS metabolome detection platform. A total of 745 metabolites were detected, among which terpenes (120, 16.11%), flavonoids (102, 13.69%), and fatty acids (160, 21.48%) are the main metabolites. The secondary metabolites detected include some with proven medicinal functions, such as genistin, Chikusetsusaponin IVa, and Pipecolic acid. PCA and OPLS-DA showed independent regions on both score plots for the five hyacinth bean varieties, indicating that the color of the seed coat and the region of origin have a significant impact on the seeds’ metabolic characteristics. Using a *p*-value < 0.05 and VIP ≥ 1 as criteria, the metabolites identified were found to be mainly secondary metabolites. White hyacinth beans were found to have higher levels of phenolic acids, coumarins, and terpenes, while red hyacinth beans have higher levels of flavonoids. Compared to the other varieties, Chongming hyacinth white bean has a higher medicinal value, and glycitin, ginsenoside Ro, diferuloyl glycerol, isopongflavone, procyanidin B2, and pratensein are speculated to be its characteristic metabolites. DPPH and FRAP assays showed that the antioxidant activity of Chongming white hyacinth bean seeds was significantly higher than that of the other four varieties. Based on the results of K-means clustering analysis and antioxidant analysis, 109 metabolites were found to have consistent content change trends in terms of antioxidant activity. Among them, 11 metabolites were confirmed to have antioxidant activity. Therefore, it is speculated that these 11 metabolites are key antioxidant substances in hyacinth bean seeds. This study enriches our knowledge of the metabolites in hyacinth bean seeds, providing a vital reference for hyacinth bean cultivation according to local conditions to improve quality.

## Figures and Tables

**Table 1:** The number of different metabolites among five hyacinth bean seeds.

Class	SCLW vs. SLP		SCLW vs. SP		SLW vs. SLP		SLW vs. SP		YLW vs. SLP		YLW vs. SP		SLW vs. SCLW		YLW vs. SCLW		YLW vs. SLW		SLP vs. SP	
	U	D	U	D	U	D	U	D	U	D	U	D	U	D	U	D	U	D	U	D
Amino acids and Peptides	6	12	6	20	15	29	13	35	11	28	12	32	15	34	11	31	10	10	1	8
Carbohydrates	6	2	9	3	6	7	8	6	9	4	8	7	4	9	5	7	1	0	1	1
Fatty acids	25	10	15	10	20	32	18	34	20	25	25	33	12	41	22	36	12	11	5	5
Phenolic acids	17	11	17	14	17	9	17	17	18	13	19	18	18	14	17	13	7	4	3	13
Flavonoids	32	25	30	29	15	62	15	62	19	53	17	59	21	52	28	44	39	8	3	9
Lignans and Coumarins	15	6	12	2	16	11	16	8	13	4	13	7	11	13	13	9	6	5	1	4
Isoflavonoids	4	3	3	3	6	4	7	4	6	3	6	4	7	2	6	3	2	3	2	2
Terpenoids	38	7	27	15	36	21	33	27	43	19	36	28	28	36	34	33	16	11	4	16
Alkaloids	11	6	10	11	16	16	12	18	16	12	15	16	12	18	13	14	5	4	0	4
Polyketides	7	2	3	2	5	8	6	10	5	7	6	9	6	10	7	9	6	4	2	1
Other	18	6	16	9	11	14	8	22	12	12	11	14	7	19	9	16	6	5	2	7

## Data Availability

The original contributions presented in the study are included in the article/Supplementary Materials, further inquiries can be directed to the corresponding author.

## Supplementary Materials

The following supporting information can be downloaded at: https://www.mdpi.com/article/10.3390/foods14111939/s1, Table S1: Environment Characterization information of four quinoa Origin areas; Table S2: The metabolomics data of five hyacinth beans seeds; Table S3: The overlapping different metabolites in SCLW vs. SLP and SCLW vs. SP with Fold Change > 10; Table S4: The overlapping different metabolites in SCLW vs. YLW and SCLW vs. SLW with Fold Change > 10; Table S5: The differential metabolites similar content change trends with antioxidant activity among five hyacinth beans seed; Figure S1: Total Ion Chromatograms (TICs) in positive (A) and negative (B) ionization modes of five hyacinth bean samples.
