# Supplementary material for: Comparing the Metabolic Characteristics of Hyacinth Bean (Lablab purpureus L.) Seeds from Five Local Varieties by UHPLC-QE HF HRMS
Source: Foods. 2025 May 29;14(11):1939. doi: 10.3390/foods14111939 (PMC12154551; doi:10.3390/foods14111939)
Supplement: Supplementary file 1 [file foods-14-01939-s001.zip › foods-3626359-supplementary/supplementary files/Figure S1.pdf]

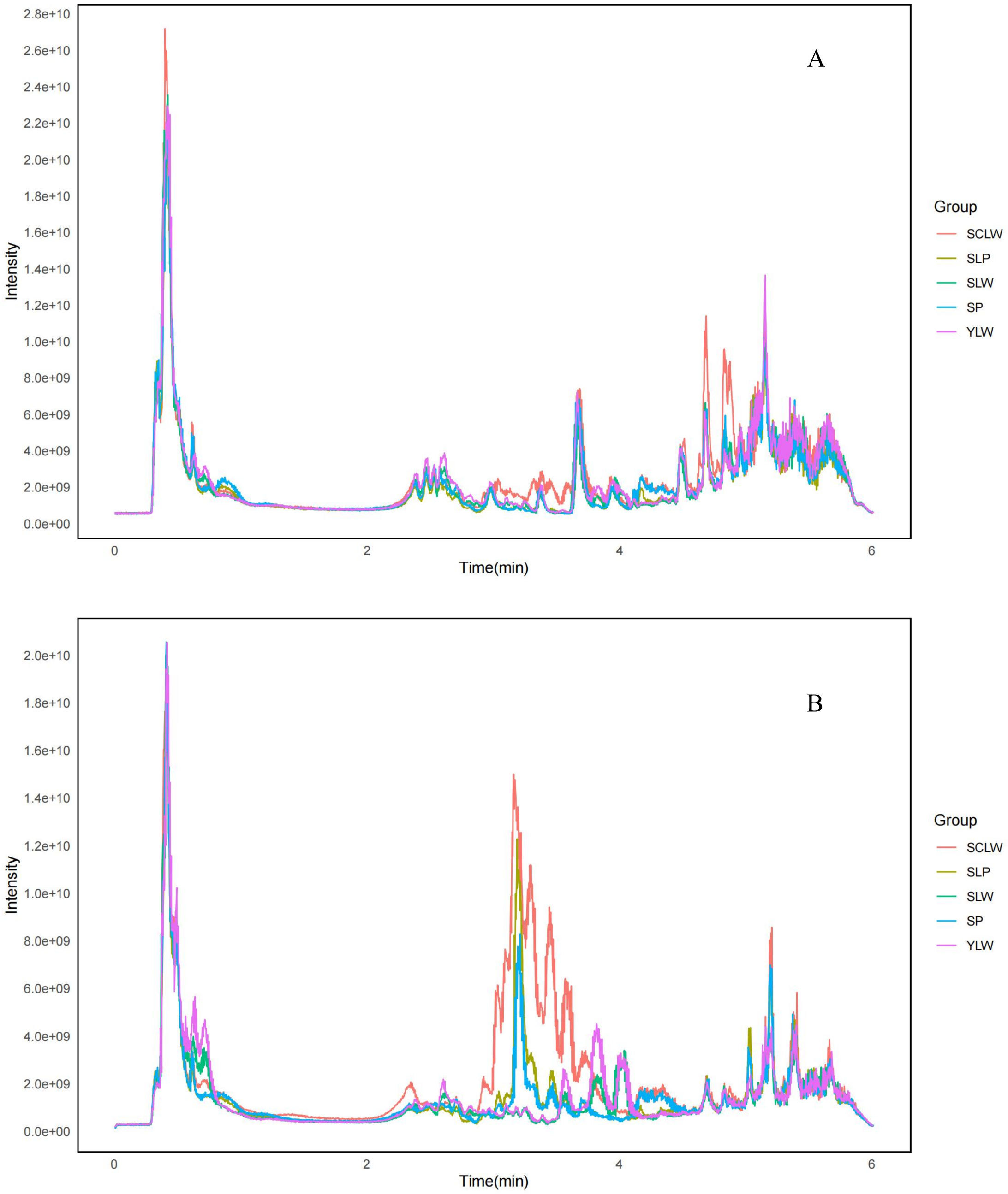

Figure S1 Total Ion Chromatograms (TICs) in positive(A) and negative(B) ionization modes of five hyacinth bean samples
