# Supplementary material for: Comparing the Metabolic Characteristics of Hyacinth Bean (Lablab purpureus L.) Seeds from Five Local Varieties by UHPLC-QE HF HRMS
Source: Foods. 2025 May 29;14(11):1939. doi: 10.3390/foods14111939 (PMC12154551; doi:10.3390/foods14111939)
Supplement: Supplementary file 1 [file foods-14-01939-s001.zip › foods-3626359-supplementary/supplementary files/Table S1 Environment Characterization information.pdf]

Table S1 Environment Characterization information of five hyacinth beans Origin areas

Origin	Geographical location	Planting area Altitude （m）	The annual average temperature (℃)	Climate	Average annual rainfall （mm）
Chongming District, Shanghai	121°09″E, 31°27′N	3.5	16.5	Subtropical monsoon climate	1025
Pudong District, Shanghai	121°27″E, 30°53″N	3.87	18.8	Subtropical monsoon climate	1100
Jiading District, Shanghai	121°26″E, 31°39′N	3.3	17.6	Subtropical monsoon climate	1077
Shuangbai County, Yunnan Province	101°03E, 24°13′ N	900	15	North subtropical plateau monsoon climate	927
Yanbian County, Sichuan Province	101°08″E, 26°25″N	1000	20	South Asian subtropical semi-arid monsoon climate	1200
